# Supplementary material for: The study of pentagonal chain with respect to schultz index, modified schultz index, schultz polynomial and modified schultz polynomial
Source: PLoS One. 2024 Jun 18;19(6):e0304695. doi: 10.1371/journal.pone.0304695 (PMC11185496; doi:10.1371/journal.pone.0304695)
Supplement: Attachment — Submitted filename: Review-PONE-D-23-40415.docx [file pone.0304695.s001.docx]

**ID:** PONE-D-23-40415 **Title:** The study of pentagonal chain with respect to schultz index, modified schultz index, schultz polynomial and modified schultz polynomial
**Author:** Dr Shahid Zaman
**Summary:**

- Title of the article is suitable.
- The manuscript is well organized and written clearly enough to be accessible.
- The subsections and structure of the manuscripts are appropriate.
- All references are included.
