# Supplementary material for: The study of pentagonal chain with respect to schultz index, modified schultz index, schultz polynomial and modified schultz polynomial
Source: PLoS One. 2024 Jun 18;19(6):e0304695. doi: 10.1371/journal.pone.0304695 (PMC11185496; doi:10.1371/journal.pone.0304695)
Supplement: Attachment — Submitted filename: respose to the reviewer comments.docx [file pone.0304695.s002.docx]

**Response/compliance to referee comments/suggestions**

The editor,

PLOS ONE.

Subject: **Submission of revised manuscript**

Respected Editor,

We would like to express our sincere thanks to you and the reviewers for valuable comments to improve our manuscript with title **“The study of pentagonal chain with respect to schultz index, modified schultz index, schultz polynomial and modified schultz polynomial”**.

We have read the comments carefully and revised the manuscript by following the reviewers' directions. The revised parts of the manuscript are colored yellow.

The detailed responses to reviewers’ comments are given below:

**Referee 1:**

**Comment 1.** Kindly discuss the real time applications on several significant distance-based topological indices for pentagonal chain graphs including the imperial index (wiener index), Gutman index, Schultz index,

modified Schultz index, Schultz polynomial, and modified Schultz polynomials on Particular example.

**Response/compliance:** Thank you very much for your invaluable suggestions. We have added the applications of the considered topological indices in the abstract and introduction. Meanwhile, we have cited some interesting articles which support to the real time applications of our main results.

**Referee 2:**

**Comment 1).** The abstract of this paper is not attractive. Please make it to the point.

**Response/compliance:** Thank you for your comment. We have revised the abstract by adding some interesting applications and focused only our contribution.

**Comment 2).** Introduction section: This section must be further enhanced by including and discussing some more latest relevant studies. Also, the authors should add some literature about “pentagonal chain”.

**Response/compliance:** We have done accordingly.

**Comment 3).** In the 1st line of introduction, it seems that the authors have denote a graph by “G=(V_G,E_G)” so in the whole manuscript they must use the notation of vertex set and edge set according to this.

**Response/compliance:** We have done accordingly.

**Comment 4).** In the second paragraph of introduction, the authors have used some notations without defined them. Similar, problem appears in many places of this paper. Please define all the notations and terminologies at first and then use them.

**Response/compliance:** We have done accordingly.

**Comment 5).** Page#4: Please revise the sentence: “Their indices' Schultz polynomial.”

**Response/compliance:** We have revised accordingly.

**Comment 6).** In the whole manuscript what is “ ” ?.

**Response/compliance:** We have defined this term.

**Comment 7).** In the Proof of theorem 4.1, the authors have skip some important mathematical steps, please include them.

**Response/compliance:** We have added those steps.

**Comment 8).** In the proof of theorem 4.1: “Point 1 of ” should be revise as “vertex 1 of :” Similar, problem repeats in the remaining proof of the theorems.

**Response/compliance:** We have revised accordingly.

**Comment 9).** I noticed that the original code/data for this article is not available. If in case the authors have developed codes for computations, it is suggested to make it open-source, its popularity can be further increased. There is no need for it if the computations are done manually.

**Response/compliance:** Thank you for your comment. Since, our results only based on mathematical calculations, we did not use any code/data.

**Comment 10).** It will further enhance the quality of this work if the current results are compared with some more latest relevant studies.

**Response/compliance:** We have done accordingly.

**Comment 11).** In the results section, add one or more paragraphs about the physical significance of their results in order to prove the importance and application their findings.

**Response/compliance:** We have done accordingly.

**Comment 12).** Conclusion needs further improvement and future research directions should be added.

**Response/compliance:** We have done accordingly.

**Referee 3:**

Use standard notation. points should be replace by vertices, don't use Greek words to represent vertices for example replace omega by n and so on. paper is not well written. Give details of proof. At the moment paper has some formulas that can hard to verify for larger values. In Table 1, some remarks were written about the finding. Explain Conclusion section need to improve, the authors need to justify claim "These metrics are employed to forecast the chemical and physical characteristics of molecules"

**Response/compliance:** Thank you very much for your invaluable suggestions. In the updated version we have replaced the word “points” with vertices, “Omega” with “n” also replaced various notations with the standard notations. We have added some more important calculations in the proofs of our theorems which will be helpful to the readers for better understanding of these mathematical results. Indeed, the formulas are for general pentagons, but it is easy to verify for one pentagon, two pentagons, and up-to so-on, as given in Table 1.

We hope that these revisions are satisfactory and that the revised version will be acceptable for publication in **PLOS ONE**. Thank you very much

Yours sincerely

Dr. Shahid Zaman
