# Supplementary material for: The study of pentagonal chain with respect to schultz index, modified schultz index, schultz polynomial and modified schultz polynomial
Source: PLoS One. 2024 Jun 18;19(6):e0304695. doi: 10.1371/journal.pone.0304695 (PMC11185496; doi:10.1371/journal.pone.0304695)
Supplement: Attachment — Submitted filename: respose to the reviewer comments.docx [file pone.0304695.s003.docx]

**Response/compliance to referee comments/suggestions**

The editor,

PLOS ONE.

Subject: **Submission of the 2^nd^ revised manuscript**

Respected Editor,

We would like to express our sincere thanks to you and the reviewers for valuable comments to improve our manuscript with title **“The study of pentagonal chain with respect to schultz index, modified schultz index, schultz polynomial and modified schultz polynomial”**.

We have read the comments carefully and revised the manuscript by following the reviewers' directions. The revised parts of the manuscript are colored yellow.

The detailed responses to reviewers’ comments are given below:

**Referee 2:**

**Comment 1.** In abstract “It also uses to predict the…” should be revised as “ It also predict the… ”

**Response/compliance:** Thank you very much for your invaluable suggestions. We have revised accordingly.

**Comment 2).** In abstract: “we consider a pentagonal…” should be revised as “ we have considered a pentagonal…”

**Response/compliance:** Thank you for your comment. We have revised accordingly.

**Comment 3).** Please add one more keyword.

**Response/compliance:** We have done accordingly.

**Comment 4).** The heading of section 3 should be revised.

**Response/compliance:** We have done accordingly.

**Comment 5).** The statement of Theorem 4.3 is not appropriate.

**Response/compliance:** We have revised it.

**Comment 6).** Add some physio chemical applications in the conclusion section.

**Response/compliance:** We have added in the revision.

We hope that these revisions are satisfactory and that the revised version will be acceptable for publication in **PLOS ONE**. Thank you very much

Yours sincerely: Dr. Shahid Zaman
